# Anticancer Activity of Fascaplysin against Lung Cancer Cell and Small Cell Lung Cancer Circulating Tumor Cell Lines

**DOI:** 10.3390/md16100383

**Published:** 2018-10-14

**Authors:** Barbara Rath, Maximilian Hochmair, Adelina Plangger, Gerhard Hamilton

**Affiliations:** Department of Surgery, Medical University of Vienna, A-1090 Vienna, Austria; barbara.rath@gmx.eu (B.R.); maximilian.hochmair@wienkav.at (M.H.); a01331326@unet.univie.ac.at (A.P.)

**Keywords:** fascaplysin, lung cancer, circulating tumor cells, signal transduction, cytotoxicity, cisplatin

## Abstract

Lung cancer is a leading cause of tumor-associated mortality. Fascaplysin, a bis-indole of a marine sponge, exhibit broad anticancer activity as specific CDK4 inhibitor among several other mechanisms, and is investigated as a drug to overcome chemoresistance after the failure of targeted agents or immunotherapy. The cytotoxic activity of fascaplysin was studied using lung cancer cell lines, primary Non-Small Cell Lung Cancer (NSCLC) and Small Cell Lung Cancer (SCLC) cells, as well as SCLC circulating tumor cell lines (CTCs). This compound exhibited high activity against SCLC cell lines (mean IC_50_ 0.89 µM), as well as SCLC CTCs as single cells and in the form of tumorospheres (mean IC_50_ 0.57 µM). NSCLC lines showed a mean IC_50_ of 1.15 µM for fascaplysin. Analysis of signal transduction mediators point to an ATM-triggered signaling cascade provoked by drug-induced DNA damage. Fascaplysin reveals at least an additive cytotoxic effect with cisplatin, which is the mainstay of lung cancer chemotherapy. In conclusion, fascaplysin shows high activity against lung cancer cell lines and spheroids of SCLC CTCs which are linked to the dismal prognosis of this tumor type. Derivatives of fascaplysin may constitute valuable new agents for the treatment of lung cancer.

## 1. Introduction

Among malignant diseases, lung cancer is the leading cause of mortality [1]. NSCLC constitutes the most common subtype with approximately 85% of cases and a 5-year survival rate ranging from 50–17%, depending on the stage of the disease [2]. SCLC accounts for the rest of the cases; it is associated with smoking and has a poor prognosis upon dissemination [3]. NSCLC tumors feature a similar poor prognosis, except for those variants amenable to specific therapies directed to mutated epidermal growth factor receptor (EGFR), anaplastic lymphoma kinase (ALK), and other kinases [4,5]. Targeted therapies in the form of tyrosine kinase inhibitors (TKIs) and immunotherapy directed to checkpoint proteins have successfully changed the treatment of NSCLC; however, patients lacking markers for precision medicine or eventually progressing after specific regimens are nevertheless referred to classical chemotherapy consisting of platinum-drug-based combinations [6]. Cisplatin/carboplatin combined with either etoposide, docetaxel, or pemetrexed have limited clinical activity, and new agents may lead to increased responses and survival. The dismal prognosis of SCLC seems to be linked to the formation of large spheroidal aggregates, termed tumorospheres, which are difficult to eliminate due to poor drug perfusion and to the existence of quiescent and hypoxic tumor cells in the interior layers of the 3D-structures [7]. A host of diverse drugs have failed to provide clinical improvements for SCLC in recent decades [3].

**Figure 1:**
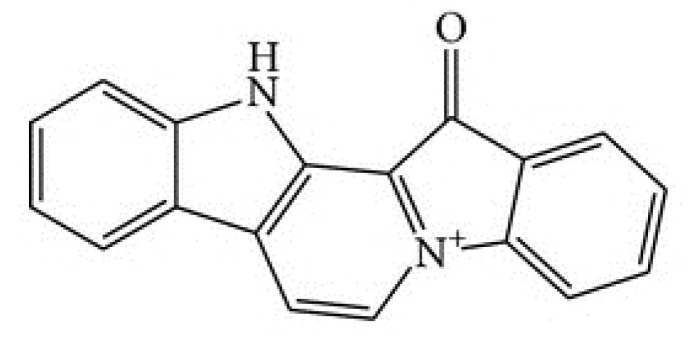
Structure of fascaplysin.

The marine drug fascaplysin (12,13-Dihydro-13-oxopyrido[1,2-a:3,4-b′] diindol-5-ium chloride) is a red bis-indole alkaloid of the *Fascaplysinopsis Bergquist* sp. sponge which was isolated by Roll et al. in 1988 [8]. The structure of fascaplysin is shown in Figure 1. Novel derivatives comprise 3-bromo-fascaplysin, 4-chloro-fascaplysin, and 7-phenyl-fascaplysin, among others. Fascaplysin possesses antibacterial, antifungal, and antiviral properties as well as antiangiogenic and antiproliferative activity against a range of cancer cell lines [9,10,11]. Cyclin-dependent kinase 4 (CDK4) was reported as the main target of fascaplysin (IC_50_ of 0.35 μM), and accordingly, drug-treated cancer cell lines arrested preferentially in the G0/1 cell cycle phase [12,13,14]. Minor activity of fascaplysin was observed against other CDKs with IC_50_ of >100 μM for CDK1, >50 μM for CDK2 as well as 20 μM for CDK5 [14]. In addition, fascaplysin was demonstrated to exhibit DNA-intercalating capability with an affinity similar to those of other typical DNA intercalators [15]. Non-planar derivatives of fascaplysin have been developed in order to possibly reduce non-CD4-mediated cytotoxic effects [16].

Cytotoxicity tests showed broad activity of fascaplysin towards a panel of 36 cancer cell lines (IC_50_ values 0.6–4 μM) [9]. Anticancer activities of fascaplysin in cell lines in vitro resulted in reduced expression of CDK4, cyclin D1 and downregulation of CDK4-specific Ser795 retinoblastoma (Rb) phosphorylation in HeLa cells [17]. Fascaplysin-induced apoptosis was characterized by the activation of effector caspases, relocalization of cytochrome c into cytosol, and reduced expression of Bcl-2. Cytotoxicity of fascaplysin in chemosensitive promyelocytic HL-60 cancer cells activated both pro-apoptotic events like PARP-1 cleavage/caspase activation and triggered autophagy, as shown by the increased expression of LC3-II, ATG7 and beclin [17]. In experimental animal models, fascaplysin suppressed tumor growth in a murine sarcoma S180 through apoptosis as well as antiangiogenesis, and HCT-116 colon tumors showed reduced size in the absence of drug toxicity [18]. Angiogenesis was blocked by fascaplysin by the inhibition of vascular endothelial growth factor (VEGF) and apoptosis of endothelial cells [19].

SCLC responds to first-line chemotherapy with platinum-based drugs/etoposide, but relapses early with topotecan remaining as the single approved therapeutic agent [3]. We have previously assessed cytotoxic activity of fascaplysin against SCLC cell lines, not covered by the NCI60 cell line panel, a tumor entity that accounts for a significant fraction of lung cancer deaths [20]. Fascaplysin was found to show high cytotoxicity against SCLC cells and to induce cell cycle arrest in G1/0 at lower and S-phase at higher concentrations, respectively. The compound generated reactive oxygen species (ROS) and induced apoptotic cell death in the chemoresistant NCI-H417 SCLC cell line. Furthermore, fascaplysin revealed marked synergism with camptothecines [21,22]. Fascaplysin IC_50_ values measured in SCLC cell lines were found to be similar to the two chemoresistant NSCLC cell lines H1299 and A549 and the chemosensitive H23 cell line, respectively.

In the present work, the investigation of the cytotoxic effects of fascaplysin is extended to include single cell suspensions and spheroids of SCLC circulating tumor cells (CTCs) and several NSCLC cell lines. Our lab has established a panel of 6 CTC SCLC cell lines derived from the blood samples of distinct patients with extended disease SCLC [7]. Furthermore, the effects of fascaplysin on the main pathways of cellular signal transduction and stress response were assessed employing phosphoprotein Western blot arrays and the NCI-H526 SCLC and the A549 NSCLC cell line, respectively.

## 2. Results

### 2.1. Fascaplysin Cytotoxicity against SCLC, NSCLC and Non-lung Cancer Cell Lines

**Figure 2:**
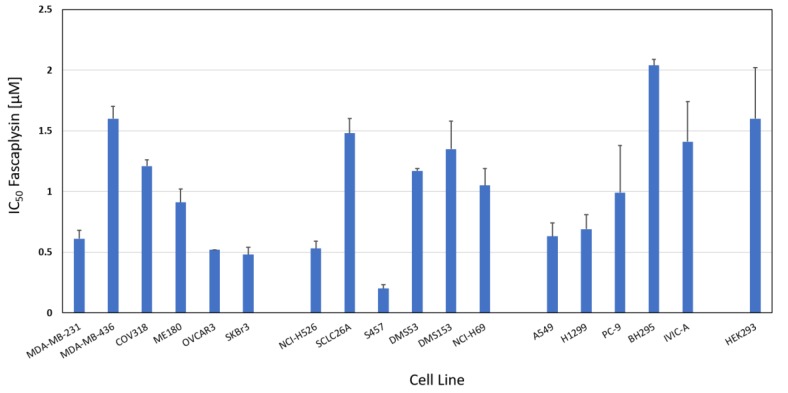
Fascaplysin chemosensitivity of a panel of SCLC, NSCLC and a panel of non-lung cancer lines. IC_50_ values are presented as mean values ± SD. Non-lung cancer cells used for comparison are breast and ovarian cancer cell lines and nonmalignant HEK293 cells are shown as normal tissue control.

The chemosensitivity of a range of cancer cell line to fascaplysin was measured in MTT cytotoxicity assays. Figure 2 shows the IC_50_ values of breast cancer and ovarian cell lines (range: 0.48–1.21 µM), SCLC cell lines (range: 0.2–1.48 µM) and NSCLC cell lines (range: 0.63–2.04 µM). Whereas SCLC and breast/ovarian cancer cell lines exhibited similar mean IC_50_ values (0.96 ± 0.5 versus 0.89 ± 0.45 µM), NSCLC cell lines proved to be less sensitive (1.15 ± 0.59 µM). SCLC26A and S457 are primary SCLC cell lines derived from pleural effusions of patients before and after therapy failure, respectively. The nonmalignant HEK293 cell line showed an IC_50_ value of 1.6 ± 0.42 µM. BH295 and IVIC-A are primary NSCLC cell lines derived from pleural effusions of patients with ALK and EGFR TKI resistance. Numrical values of the IC50 data are presented in Supplementary Table S1.

### 2.2. Fascaplysin Cytotoxicity against SCLC CTC Single Cells and Tumorospheres

**Figure 3:**
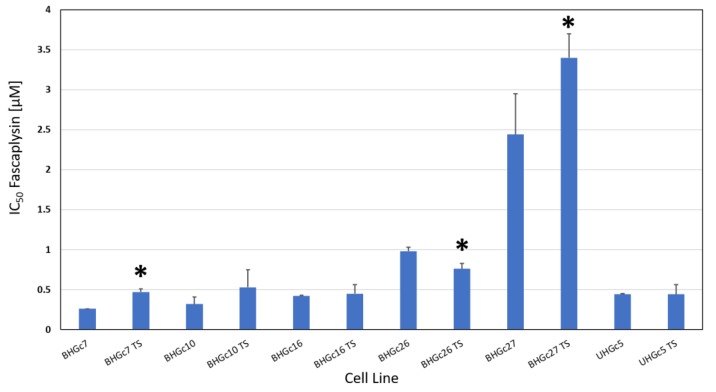
Chemosensitivity of SCLC CTC lines for fascaplysin. The CTC lines were tested in form of single cell suspensions and as tumorospheres. IC_50_ values are presented as mean values ± SD and significant differences between 2D- and 3D-cultures are indicated by an asterisk.

The SCLC CTCs form spontaneously large spheroids which are markedly chemoresistant to cisplatin and other drugs used for the treatment of SCLC patients in comparison to CTCs in form of single cell suspensions. The chemosensitivity of such single cell suspensions and tumorospheres against fascaplysin were compared in MTT tests (Figure 3). With exception of BHGc26 and 27 CTC lines, fascaplysin IC_50_ values of the other lines were equal or below 0.5 µM. A comparison of the ratios of IC_50_ values of single cell suspensions and tumorospheres for cisplatin and fascaplysin demonstrates that for fascaplysin, the differences in chemosensitivities between these 2D- and 3D-cultures are much less than for the platinum drug (Table 1) indicating superior anticancer activity for spheroids.

Fascaplysin versus cisplatin showed a 1.5 fold increased cytotoxic activity for tumorospheres for BHGc10 and BHGc27, 2.5 fold for BHGc7 and UHGc5, and 6.7 fold for BHGc16 and 26, respectively (Table 1). The mean cytotoxicity ratios between fascaplysin and cisplatin are significantly different for all SCLC CTC cell lines.

### 2.3. Alterations of Selected Phosphoproteins of NCI-H526 and A549 in Response to Fascaplysin

**Figure 4:**
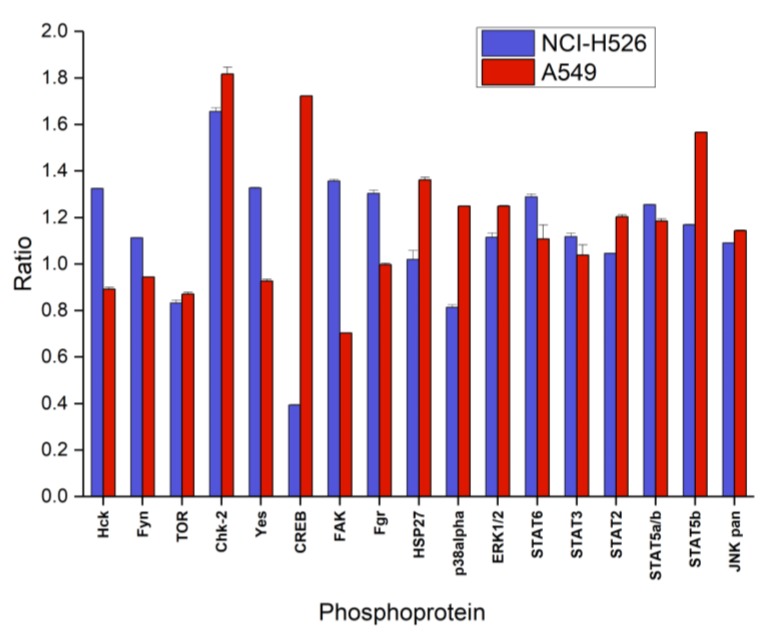
Relative phosphorylation (ratio of treatment:control) of selected components (part A of the array) of the signal transduction system (mean ± SD) of NCI-H526 and A549 cells treated with 0.5 µM fascaplysin for 72 h (NCI-H526: significantly different to controls, except for HSP27 and STAT2; A549 significantly different to controls, except Fgr and STAT3).

Figure 4 shows the first part of the phosphoproteins assayed with the ARY003 human proteome profiler kit for fascaplysin-treated NCI-H526 and A549, respectively. In contrast to the cytotoxicity assays, incubation time for phosphoprotein analysis was reduced to 72 h to prevent cell death. In NCI-H526 SCLC cells fascaplysin induced significantly increased phosphorylation of src kinases (Hck, Fyn, Yes and Fgr), CHK-2 and FAK, whereas phosphorylation of mTOR, CREB and p38α was significantly decreased compared to untreated controls. In contrast, A549 NSCLC cells revealed increased phosphorylation of CHK-2 in combination with CREB, HSP27, and STAT5b, with decreased phosphorylation of src kinases (except Fgr) and FAK.

**Figure 5:**
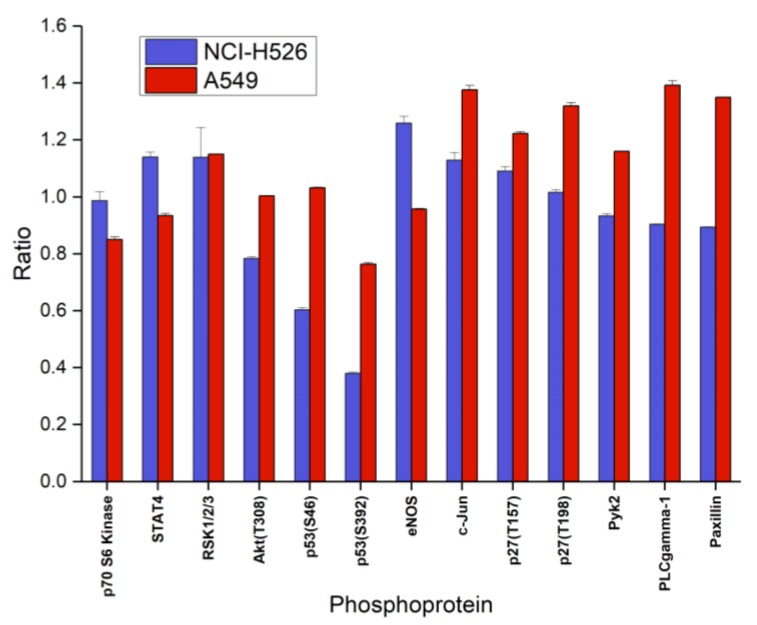
Relative phosphorylation (ratio treatment: control) of selected components (part B of the array) of the signal transduction system (mean ± SD) of NCI-H526 and A549 cells treated with 0.5 µM fascaplysin for 72 h (NCI-H526: significantly different to controls, except forp70 S6 kinase and p27; A549 significantly different to controls, except Akt, p53/S46, and eNOS).

Analysis of the second part of phosphoproteins of the ARY003 kit yielded decreased phosphorylation of Akt, p53(S46/S392) and increased phosphorylation of STAT4, eNOS, c-Jun, and p27(T157) in the case of fascaplysin-pretreated NCI-H526, and numerous increases of phosphorylation in A549 cells, with the exception of decreases in p70 S6 kinase, STAT4, and p53(S392) (Figure 5). Phosphoproteins of the ARY003 blots which exhibited no significant changes for NCI-H526 or A549 cells in response to treatment with fascaplysin were not included in these figures.

### 2.4. Signaling Pathways Affected by Fascaplysin in NCI-H526 and A549 Lung Cancer Cells

**Figure 6:**
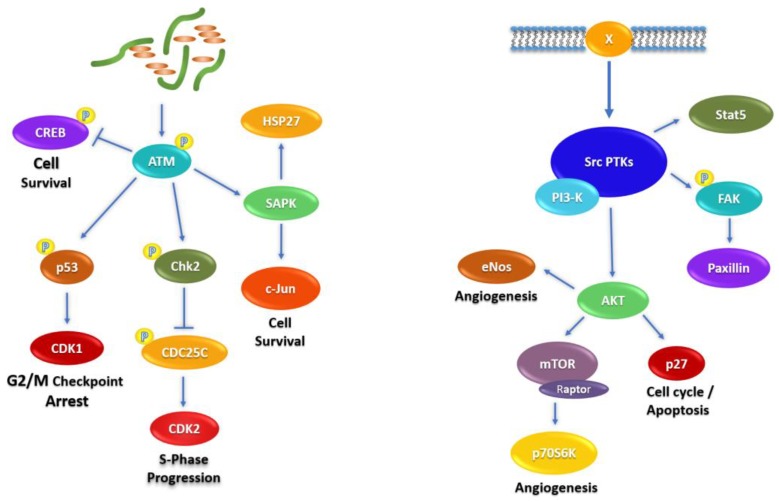
Schematic presentation of the signaling pathways involved in fascaplysin treatment of NCI-H526 and A549 lung cancer cells.

The signal transduction mediators related to fascaplysin-induced alterations in NCI-H526 and A549 cells are depicted schematically in Figure 6. The schemes start with fascaplysin-induced DNA damage (left) and receptors/src kinases (right), respectively. Chk2 is activated by upstream DNA damage-sensing ATM and modulate functions of CREB, p53, CDC25, and stress kinases (left). Src kinases are activated by a number of connected membrane receptors (X) or oncogenic mutation, and regulate the activities of Stat5, FAK, and the Akt–mTOR axis (right).

### 2.5. Combination of Cisplatin/Etoposide with Fascaplysin in Cytotoxicity Assays for NCI-H526 and A549 Cell Lines

**Figure 7:**
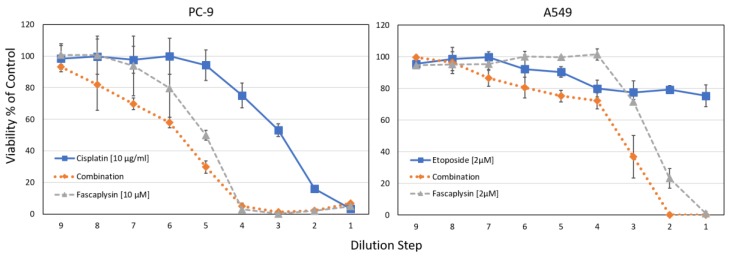
Cytotoxicity tests investigating fascaplysin combinations with chemotherapeutics.

Combination indices (CI) were calculated using the Chou-Talaly method, indicating synergy at values <1 [23]. For NCI-H526, CIs < 0.49 were found for cisplatin concentrations 0.625–5 µg/mL and 0.125–1 µM fascaplysin (fixed ratio of 2.5:1), similar to A549 with CI < 0.62 for cisplatin concentrations 1.25–10 µg/mL and 0.25–2 µM fascaplysin (fixed ratio of 5:1). For the NSCLC lines PC-9 and A549, the synergistic effects of fascaplysin with cisplatin or etoposide are shown in Figure 7.

For the NSCLC cell lines PC-9 and A549, combinations of fascaplysin and cisplatin or etoposide were tested in cytotoxicity tests. For both tests, IC_50_ values for the combinations using the concentrations as indicated revealed synergistic interactions. The CI values ranged from 0.26–0.76 for PC-9 fascaplysin-cisplatin and from 0.1–0.94 for A549 fascaplysin-etoposide, respectively. Data are shown as mean ± SD, the initial concentrations were titrated in 9 two-fold dilution steps.

## 3. Discussion

Lung cancer is the leading cause of cancer-related mortality in both men and women worldwide [1]. Targeted therapy is applicable to a minor fraction of NSCLC patients [4]. Patients with advanced lung cancer exhibit low survival rates and novel modes of chemotherapy need to be developed [2]. Deregulated proliferation of tumor cells is accomplished by alterations of the cell cycle and checkpoint controls amenable to inhibition by targeting of cell cycle and checkpoint kinases (CDKs) [24]. In particular, CDK4/6 inhibitors seem to present suitable targets in a majority of patients with advanced cancer [25,26]. Besides CDK4/6 inhibitors palbociclib and LY2835219, which have shown high activity in breast cancer, a host similar drugs are under development, and fascaplysin and derivatives share the same target [27]. Proteins in this cell proliferative pathway include p16, an endogenous suppressor of CDK4/6, cyclin D1, the regulatory subunit of CDK4/6, and retinoblastoma (Rb) protein, a tumor suppressor [28]. Both CDK4 and CDK6 encode cyclin-dependent kinases which complexed with cyclines of the D-type phosphorylate the Rb protein. Rb in turn triggers the expression of gene products for G1-S phase cell cycle progression. Rb inactivation is a common event in lung cancer, and is more frequent in SCLC than in NSCLC [29]. In SCLCs, Rb alterations can be found in a high percentage of cases, i.e., from 88% to 100% of the biopsy samples [30]. Therefore, in the present study we compared the effects of fascaplysin in the A549 Rb-wildtype NSCLC cell line to the Rb-mutated NCI-H526 SCLC cell line. Although, both cell lines have a similar chemosensitivity to fascaplysin, analysis of the intracellular signal transduction by Western blotting of selected phosphoproteins revealed marked differences in response to this drug.

DNA damage response is triggered when sensor proteins ATM (ataxia telangiectasia mutated) and ATR (also called ataxia telangiectasia and Rad3-related protein) detect structural distortions or breaks [31]. After DNA damage, CHK2 is phosphorylated by ATM on the priming site T68, and in turn, phosphorylates more than 24 proteins to induce apoptosis, DNA repair, or tolerance of the damage [32]. In wildtype cells, CHK2 phosphorylates Rb which enhances the formation of the transcriptionally-inactive pRb/E2F-1 complex causing G1/S arrest and suppression of apoptosis. Pronounced activation of CHK-2 in NCI-H526 and A549 cells indicates direct damage of DNA by fascaplysin and activation of the corresponding cellular responses in both cell lines. The cyclic AMP response element-binding protein (CREB) initiates transcriptional responses associated with cell survival to a wide variety of stimuli following its phosphorylation on Ser-133. Whereas fascaplysin treatment resulted in decreased phosphorylation of CREB in NCI-H526 cells, this transcription factor is hyperphosphorylated in A549 cells, possibly indicating anti- and pro-survival signaling, respectively [33,34]. Furthermore, cisplatin-induced activation of FAK has been linked to increased chemoresistance in ovarian cancer cells and FAK inhibitors induce tumor cell apoptosis [35]. Activated FAK forms a complex with Src family kinases and seems to provide a prosurvival signal in NCI-H526 cells, in contrast to fascaplysin-treated A549 cells [36]. In addition, overexpression of Src in cancer accelerates metastasis and is responsible for chemoresistance via multiple downstream signaling pathways, concerning Akt, MAPKs, STAT3, cytokines, etc. [37]. Therefore, activation of a number of Src kinases in NCI-H526 cells (Hck, Fyn, Yes and Fgr) may counteract fascaplysin toxicity and retard cell death; possibly contributing to the observed slower rate of loss of viability in the presence of increasing doses of fascaplysin in these cells. The stress kinases p38 and JNK are generally activated by inflammatory cytokines and different stressors, including DNA-damaging compounds [38]. p38 MAPK signaling results in the phosphorylation of CREB at Ser133, which seem to occur in A549 cells, contrary to NCI-H526 which shoes decreased p38 activity and phosphorylation of CREB [39]. Clearly, fascaplysin is an inhibitor of CDC25, and this pathway is expected to be inhibited in NCI-H526 cells [40].

In conclusion, fascaplysin shows marked anticancer activity in NSCLC and SCLC cells independently of the function of the CDK4 pathway, thus pointing to direct effects on DNA and the transcription of various proteins. The mechanisms of the antitumor effect of fascaplysin demonstrated on several carcinoma models indicate that fascaplysin is close to some drug groups such as intercalating agents, inhibitors of serine-threonine, and tyrosine kinases. Additionally, fascaplysin increases phosphorylation of AKT/PKB and adenosine monophosphate-activated protein kinase (AMPK), which feature anti-apoptotic or pro-survival functions in cancer [41]. In detail, fascaplysin abolishes the phosphorylation of mTOR, 4EBP1, and p70S6K1, which trigger the cap-dependent translation machinery and affect the expression of oncoproteins, such as survivin, c-myc, cyclin D1, VEGF, and HIF-1α. Similarly, 7-chloro-fascaplysin inhibited cell survival through interference with the PI3K/Akt/mTOR pathway, which in turn modulates HIF-1α, eNOS and MMP-2/9 in a breast cancer cell line [42]. The cytotoxicity of 4-chlorofascaplysin (4-CF) was reversed by co-treatment with the VEGF and Akt inhibitors or in response to neutralizing VEGF antibodies. Fascaplysin has stronger anti-cancer effects than other CDK4 inhibitors on lung cancer cells that are wild-type or null for Rb, indicating that unknown target molecules might be involved in the antitumor activity of fascaplysin [43]. In good accordance with the results of Oh et al. and Sharma et al., our results show alterations of phosphoproteins altering the Akt-mTor pathway which are triggered mainly by upstream stress and src kinases.

Relapsed SCLC is resistant to a wide range of drugs and clinical trials have not led to improvements in survival rates over recent decades. Chemoresistance of SCLC seems to be related to the formation of large spheroids, termed tumorospheres, which limit drug access and contain quiescent and hypoxic tumor cells which are less sensitive to chemotherapeutics. Such 3D-structures were demonstrated to show increased resistance to cisplatin, etoposide, topotecan, and epirubicin when compared to the same SCLC CTC cells in form of single cell suspensions. In particular, fascaplysin is cytotoxic against SCLC CTC tumorospheres which exhibit high chemoresistance against a range of commonly-administered chemotherapeutics. Fascaplysin-induced cell death of outer SCLC CTC cell layers seems to trigger the elimination of the whole spheroid. Especially in SCLC cells, the induction of ROS by fascaplysin is expected to exert increased damage due to the small volume of the cytoplasmic fraction [7]. It should be noted that spheroids are similarly observed in pleural effusions of NSCLC patients. Although the parent drug fascaplysin seems too toxic for clinical application, derivatives such as 3-bromofascaplysin and 7-phenylfascaplysin were demonstrated to possess higher cytotoxic efficiency and different profiles [44,45,46]. Furthermore, the alkaloid derivative 4-CF exhibits five times higher cytotoxic IC_50_ value in normal cells, as well as no apparent toxicities in murine xenograft models at therapeutic doses [42].

## 4. Cell Culture and Methods

### 4.1. Chemicals

Unless otherwise noted, all chemicals were obtained from Sigma-Aldrich (St. Louis, MO, USA). Dulbecco’s phosphate buffered saline (PBS) was purchased from Gibco/Invitrogen (Carlsbad, CA, USA). Compounds were prepared as stock solutions of 2 mg/mL in either DMSO or in 0.9% NaCl solution (cisplatin), sterilized by filtration in case of cisplatin, and aliquots stored at −20 °C.

### 4.2. Cell Culture

The A549 NSCLC A549 (Rb/p53 wild-type) and NCI-H526 SCLC A549 (Rb protein not expressed/p53 wild-type) cell lines were obtained from the American Type Culture Collection (Rockville, MD, USA), as well as the other cell lines except primary lines and all SCLC CTCs established in our lab [7]. Cells were grown in RPMI-1640 bicarbonate medium (Seromed, Berlin, Germany), supplemented with 10% FBS (Seromed), 4 mM glutamine, and antibiotics (final concentrations: 50 U/mL of penicillin, 50 µg/mL of streptomycin, and 100 µg/mL neomycin; Sigma-Aldrich, St. Louis, MO, USA), and subcultivated twice a week. A549 is p53 wildtype and DNA profiling by short tandem repeat analysis of the NCI-H526 cells proved their identity to the American Type Culture Collection specifications, and the yeast p53 functional assay revealed expression of fully active p53 (functional assay of separated alleles in yeast FASAY; data not shown).

### 4.3. Phosphokinase Array

Relative protein phosphorylation levels of 38 selected proteins were obtained by analysis of 46 specific phosphorylation sites using the Proteome Profiler Human Phospho-Kinase Array Kit ARY003 (R&D Systems, Minneapolis, MN, USA) in duplicate tests according to the manufacturer’s instructions. Briefly, cells were rinsed with PBS, 1 × 10^7^ cells/mL lysis buffer were solubilized under permanent shaking at 4 °C for 30 min, and aliquots of the lysates were stored frozen at −80 °C. After blocking, membranes with spotted catcher antibodies were incubated with diluted cell lysates at 4 °C overnight. Thereafter, cocktails of biotinylated detection antibodies were added at room temperature for 2 h. Phosphorylated proteins were revealed using streptavidin-HRP/chemiluminescence substrate (SuperSignal West Pico, Thermo Fisher Scientific, Rockford, IL, USA) and detection with a Molecular Imager VersaDoc MP imaging system (Bio-Rad, Hercules, CA, USA). Images were quantified using the ImageQuant TL v2005 software (Amersham Biosciences, Buckinghamshire, UK) and Microsoft Excel software (Microsoft, Redmond, WA, USA). The different Western blot membranes were normalized using the 3 calibration spots included. Signaling pathways affected by fascaplysin in NCI-H526 and A549 lung cancer cells were produced using Power Point software (Microsoft, Redmond, WA, USA).

### 4.4. Cytotoxicity Assay

Aliquots of 1 × 10^4^ cells in 200 µL medium were treated for four days with twofold dilutions of fascaplysin or cisplatin, respectively in 96-well microtiter plates in quadruplicate (Greiner, Kremsmuenster, Austria). For SCLC CTC tumorospheres, an equivalent number of cells in form of spheroids were tested as described [7]. The plates were incubated under tissue culture conditions, and cell viability was measured using a modified MTT (3-(4,5-dimethylthiazol-2-yl)-2,5-diphenyltetrazolium bromide) assay (EZ4U, Biomedica, Vienna, Austria). Optical density was measured using a microplate reader at 450 nm with an empty well as reference. Values obtained from control wells containing cells and media alone were set to 100% proliferation. For the assessment of the interaction of fascaplysin with cisplatin, tests were performed comprising the individual drugs alone and in combination, followed by analysis using the Chou-Talalay method with help of the Compusyn software (ComboSyn, Inc. Paramus, NJ, USA).

### 4.5. Statistics

Statistical analysis was performed using Student’s *t* test for normally distributed samples (* *p* < 0.05 was regarded as statistically significant). Values are shown as mean ± SD.

## 5. Conclusions

CDKs are a group of serine/threonine kinases which are critical in the regulation of the cell cycle. A major role of CDK-4 is the phosphorylation of Rb, which is inhibited by fascaplysin and a range of other compounds. Mutations in Rb, along with those of cyclin D and p16(INK4a), has been seen frequently during tumorigenesis of cancers. Investigation of a part of the kinome of NCI-H526 SCLC and A549 NSCLC cell lines reveals different responses to treatment with fascaplysin, most likely to be connected to the Rb phenotype. In NCI-H526 cells, fascaplysin sensitivity is determined by the absence of the CDK4–Rb pathway and DNA damage in combination with putative CDC25 inhibition, whereas in A549, inhibition of CDK4 seems to be the major effect with distinct and small effects on phosphoproteins. Fascaplysin exhibits marked anticancer activity against permanent and primary SCLC and NSCLC cells, with cytotoxic effects against SCLC CTC tumorospheres that are far superior to those of other therapeutics. Therefore, fascaplysin and derivatives with a better clinical profile may constitute valuable agents for lung cancer therapy.

## Figures and Tables

**Table 1:** Mean values of IC_50_ ratios for SCLC CTC tumorospheres versus single cell suspensions for fascaplysin and cisplatin, respectively (mean values ± SD). All ratios for fascaplysin, cisplatin, and the CTC lines are significantly different.

CTC Cell Line	Fascaplysin		Cisplatin	
	Mean Ratio (TS/SC)	SD	Mean Ratio (TS/SC)	SD
BHGc7	1.83	0.1	4.31	0.2
BHGc10	1.63	0.2	2.32	0.4
BHGc16	1.06	0.1	7.22	0.3
BHGc26	0.77	0.1	5.20	0.3
BHGc27	1.39	0.5	2.17	0.2
UHGc5	0.99	0.1	4.8	1.0

## Supplementary Materials

The following are available online at http://www.mdpi.com/1660-3397/16/10/383/s1, Table S1: Fascaplysin activity against a panel of cell lines.
