# Supplementary material for: Anticancer Activity of Fascaplysin against Lung Cancer Cell and Small Cell Lung Cancer Circulating Tumor Cell Lines
Source: Mar Drugs. 2018 Oct 14;16(10):383. doi: 10.3390/md16100383 (PMC6213142; doi:10.3390/md16100383)
Supplement: Supplementary file 1 [file marinedrugs-16-00383-s001.pdf]

Cell Line	IC_{50} Fascaplysin [µM]	SD
MDA-MB-231	0,61	0,07
MDA-MB-436	1,6	0,1
COV318	1,21	0,05
ME180	0,91	0,11
OVCAR3	0,52	
SKBr3	0,48	0,06
NCI-H526	0,53	0,06
SCLC26A	1,48	0,12
S457	0,2	0,03
DMS53	1,17	0,02
DMS153	1,35	0,23
NCI-H69	1,05	0,14
A549	0,63	0,11
H1299	0,69	0,12
PC-9	0,99	0,39
BH295	2,04	0,05
IVIC-A	1,41	0,33
HEK293	1,6	0,42
